# Psychiatry

**Published:** 1905-04-01

**Authors:** 


### PSYCHIATRY.

**Amaurotic Idiocy of the Family Type.**—Dr. James Burnet, of Edinburgh, gives an account [1] of the rare form of disease known as amaurotic family idiocy, first described by Sachs, of New York, in 1887. The disease is essentially an idiocy, or arrest of cerebral and mental development, occurring in the first year of life, and accompanied by blindness. The case recorded by Dr. Burnet was that of a male infant, aged 18 months. His parents were Jews. So far as could be made out none of their relatives had suffered from mental disease, nervous affections, or syphilis. The mother was a nervous woman who had a great deal of worry during her pregnancy. The patient was her seventh child, and was a normal full-term infant. Her previous pregnancies and labours were uneventful, and there were no miscarriages. Of her seven children, the first, a girl, died at three weeks, of facial erysipelas. The second is alive and healthy at the age of 14 years, though when an infant of 18 months she developed blindness, lasting about a year and a half, which then disappeared after an attack of measles. The fourth, a girl, gradually developed blindness, and died at 13 months of age. The fifth, a boy, is healthy and alive in his seventh year. The sixth died when a few weeks old of "fits during an attack of bronchitis." The patient, who is the seventh child, was apparently healthy. He was breast-fed until he was 14 months old. Mentally he was defective, the only words he could say were "mam" and "popo." He could not walk or sit up. The head was large, the fontanelle still open, the abdomen was very prominent, and all the epiphyses showed distinct enlargement. When three months old he had pneumonia, and suffered from bronchitis (rachitic) ever since. He developed blindness which gradually increased after the age of six months. Ophthalmoscopic examination showed atrophy of the optic discs. "Knowing as I did the family history," says Dr. Burnet, "I feared lest he too might develop blindness." The child died at the age of eighteen months in a condition of helpless idiocy, with complete blindness. "The case was evidently one of amaurotic family idiocy, and from the histories of the seven children given above it is evident that practically every second child of the family developed blindness." The disease, concludes Dr. Burnet, usually proves fatal before the age of two years, and it is a curious fact that nearly all the recorded cases have occurred in Jewish families. "I am convinced that it is not of syphilitic origin, but in all probability is due to some defect of development of the nerve tissues." Professor James Mackie, of the Philadelphia Polyclinic, also reports an interesting case of amaurotic family idiocy.[2] The patient was a year old. The mother was healthy. There was no history of insanity, tuberculosis or syphilis on the father's or mother's side. The child was born at full term, and was considered a normal baby. It suffered always from constipation, and took cold easily. The head was square, rachitic, and showed signs of craniotabes. with a widely open fontanelle. There were marked lumbar curve, "beading" of the ribs, and enlargement of liver and spleen. He could not support his head, sit upright, or stand. He was mentally deficient, and though under treatment his bodily symptoms improved, he never learnt to speak. During the second year he developed blindness. The child "took no notice of his surroundings, and did not use his arms for grasping," said the mother. Ophthalmoscopic examination showed the characteristic retinal lesions of amaurotic idiocy. The nervous symptoms included paresis of the neck muscles, so that the head rolled about when the child was moved, a spastic condition of the lower and upper limbs, and "claw-hand." The great toe was extended, and the other toes flexed as in Babinski's phenomenon. The tendon reflexes were generally exaggerated, and ankle-clonus was present. The plantar reflexes were excessive, and Babinski's reflex was present. The pupils were dilated, unequal, and failed to respond to light. Slow nystagmoid movements of the eyeballs were observed. Consciousness and intelligence were at a low level, he was exceedingly somnolescent, with a respiration of 30 and a pulse of 136 per minute. The rectal temperature was 95·6° Fahr. He died in a state of adenopathy, blindness and idiocy, at the age of $2\frac{1}{2}$ years from an attack of pneumonia and bronchitis. "In amaurotic family idiocy," says Professor McKee, "the cerebral development, at first normal, ceases at a very early age." The cerebral cells degenerate and idiocy results within the first year of life. Sachs, Brudenell Carter, and modern observers have noted that nearly all cases are members of the Jewish race. "That the disease should be common in the progeny of a hard-working, self-denying race, a race in which the neuropathic element is very prominent, is intelligible." The autopsy in the present case showed besides catarrhal pneumonia "an exceedingly heavy and œdematous brain." Microscopic examination showed that the retinal ganglion cells were increased in size and globular-looking, that the Nissl bodies were absent, that the optic nerve was denuded of myelin in many of its fibres, and that the neuroglia tissue was increased—all evidences of simple degeneration. Dr. Mary Buchanan, of Philadelphia,[3] presents the above account of the microscopic appearances, and adds that "the cerebral degenerative process can be marked by examination of the eyes. These are normal at birth, at the age of three or four months there is a haziness in the macular region, and the picture of macular atrophy is complete generally in the sixth month."

[1] Journ. of Med. Science, January, 1905. [2] Amer. Jour. of the Med. Sciences, January, 1905. [3] Ibid.

*(To be continued.)*

